# A formative evaluation of the SWITCH® obesity prevention program: print versus online programming

**DOI:** 10.1186/s40608-015-0049-1

**Published:** 2015-05-03

**Authors:** Gregory J Welk, Senlin Chen, Yoon Ho Nam, Tara E Weber

**Affiliations:** Department of Kinesiology, Iowa State University, 255 Forker Building, Ames, IA 50011 USA

**Keywords:** Behavior, Childhood obesity, Intervention, School health

## Abstract

**Background:**

SWITCH® is an evidence-based childhood obesity prevention program that works through schools to impact parenting practices. The present study was designed as a formative evaluation to test whether an online version of SWITCH® would work equivalently as the established print version.

**Methods:**

Ten elementary schools were matched by socio-economic status and randomly assigned to receive either the print (n = 5) or online (n = 5) version. A total of 211 children from 22, 3^rd^ grade classrooms were guided through the 4 month program by a team of program leaders working in cooperation with the classroom teachers. Children were tasked with completing weekly SWITCH® Trackers with their parents to monitor goal setting efforts in showing positive Do (≥60 minutes of moderate-to-vigorous physical activity), View (≤2 hours of screen time), and Chew (≥5 servings of fruits and vegetables) behaviors on each day. A total of 91 parents completed a brief survey to assess project-specific interactions with their child and the impact on their behaviors.

**Results:**

The majority of parents (93.2%) reported satisfactory experiences with either the online or print SWITCH® program. The return rate for the SWITCH® Trackers was higher (42.5% ± 11%) from the print schools compared to the online schools (27.4% ± 10.9%). District program managers rated the level of teacher engagement in regards to program facilitation and the results showed a higher Trackers return rate in the highly engaged schools (38.5% ± 13.3%) than the lowly engaged schools (28.6 ± 11.9%). No significant differences were observed in parent/child interactions or reported behavior change (*p*s > .05) suggesting the equivalence in intervention effect for print and online versions of the SWITCH® program.

**Conclusions:**

The findings support the utility of the online SWITCH® platform but school-based modules are needed to facilitate broader school engagement by classroom teachers and PE teachers.

## Background

Childhood obesity prevention is a priority on the public health agenda [1,2]. Numerous intervention programs have been designed and implemented to promote children’s lifestyle behaviors and most have been conducted in schools [3]. Fewer have examined the efficacy of family-based interventions on children’s behavior change and these studies have rendered less consistent findings [4]. A comprehensive review of youth activity promotion efforts conducted as part of Physical Activity Guidelines Midcourse Review highlighted the advantages of multi-component interventions over isolated education or curricular approaches [5]. A consensus document by the Institute of Medicine titled “Educating the Student Body” also emphasized the importance of adopting programming strategies that capture broader school-wide changes [6]. The advantage of these social-ecological approaches is that they have greater potential for both larger impacts and greater sustainability.

A promising program that is consistent with these recommendations is the SWITCH® obesity prevention program. SWITCH® is a multi-component, ecologically-based program designed to work through schools to reach families. The strategy capitalizes on the coordinating structure and motivation provided through a school-based program while also minimizing the burden on teachers and schools. Parents are viewed as the primary target of the SWITCH® program since they directly influence children’s current and long-term lifestyle behaviors [7]; however, schools play a key role by encouraging involvement in the program and by reinforcing the messages. The program targets three distinct behaviors known to impact obesity (i.e., physical activity, screen time and eating habits) by challenging children to “*switch what they do, view and chew”* [8]. Children complete weekly tracking charts (“SWITCH Trackers”) with the help of their parents to learn self-monitoring skills and they receive incentives through the school for their participation. Consistent with current recommendations, the overall goal is to help children obtain at least one hour of moderate-to-vigorous physical activity per day, reduce screen time to two hours or less per day, and to eat five or more servings of fruits and vegetables per day [8].

The SWITCH® program has been previously shown to be effective in promoting children’s health behaviors [9]. The original efficacy study demonstrated significant reductions in screen time, as well as increases in fruit/vegetable consumption at the post measurement and effects were retained at the 6 month follow-up measurement [9]. It was recognized as a promising program by the Let’s Move campaign [10], but the high cost of the print-based manuals and resources (~$60 per student) has limited the adoption and dissemination on a larger scale. To enhance utilization, the SWITCH® program has been converted to a web-based platform that can be delivered in a more cost-effective manner. The long term vision for the SWITCH® program is to create a robust content management system that will allow schools to effectively set up, manage and coordinate their own SWITCH® program. Schools would have access to participant and data management functions to enable customization of delivery but centralized administrative functions would ensure standardization of program delivery across multiple sites. This type of structure is consistent with recommendations for designing public health programming for effective dissemination since it can promote fidelity in implementation [11].

Experts in program dissemination emphasize the importance of systematically evaluating underlying assumptions and pre-testing different implementation methods before dissemination [12]. With SWITCH®, a fundamental question is whether the online version would have similar, worse or better outcomes than a comparable print based version. An advantage of print-based resources is that parents have immediate access to the materials, but a disadvantage (in addition to cost) is that the materials can get set aside and be ignored. Online materials are more integrated with contemporary lifestyles that revolve around email, the internet, and social media. It enables a more direct communication with parents; however, a potential disadvantage is that it is hard for online communications to consistently engage parents in a meaningful way [13,14]. Numerous studies have begun to explore the potential utility of web-based and electronic interventions [15-17] but few, if any, studies have systematically compared a print program to an online program.

The purpose of the present study was to evaluate the utility of the online SWITCH program®. To enable this work, we first developed a basic web platform (SWITCH® 1.0) which made all program-related materials available online for download (www.iowaswitch.org). The creation of this online tool made it possible to directly answer the following research question: whether or not the online version of SWITCH® would work equivalently (or better) than the existing print-based version. We used a formative evaluation plan for the study since the broader goal was to determine changes to be incorporated into the more robust web-based system envisioned for the future of the program. While the present study reports on one specific program (i.e., SWITCH®), the results have broader value for other program planners considering the relative merits of print vs online resources.

## Methods

### Design, setting and participants

Thirteen elementary schools from a large school district in a northeast Iowa completed SWITCH® programming as part of annual district programming and 10 agreed to participate in the optional SWITCH® evaluation. The school district had been running the SWITCH® program (with funding and logistical support from the local Young Men’s Christian Association [YMCA]) continuously since the original efficacy-based study back in 2006. The program has been valued in the community but the high cost has presented a barrier to sustainability and dissemination so the schools agreed to participate in a controlled evaluation of the newly developed online version of SWITCH®. Schools were matched by socio-economic status (assessed as the percent of youth qualifying for free/reduced lunch) and then randomly assigned into either the print (n = 5) or online (n = 5) versions of the SWITCH® program. The online version replicated all aspects of the print-based manual and the programming was delivered in a consistent way for both sets of schools. The matched samples and standardization of methods make it possible to directly compare the print vs online formats.

Parents voluntarily enrolled in the SWITCH® program by either returning a signed enrollment form (print) or by completing a similar online registration form (online). A total of 210 children/parent dyads enrolled in the project (print: n = 100; online: n = 110). Schools had 2–3, 3^rd^ grade classrooms involved, with total school enrollments ranging from 14 to 38. Parents from the participating schools were asked to complete a post-test survey to evaluate their engagement with and utilization perceptions of the SWITCH® program. Because the data we collected in this study were de-identified, the Iowa State University *institutional review board* (IRB) deemed the study as “exempt”, therefore, written consent from participants or their parents/guardians were also waived.

### Intervention procedures

The SWITCH® program operates over a 16 week period with new materials released each week. Programming is divided into 4 modules each following the same sequence of content: week 1 “*Switch what you DO*”, week 2 “*Switch what you VIEW*”, week 3 “*Switch what you CHEW*”, and week 4 “*You Rule! Try all 3 goals!*”. The programming in each week is enriched by activities carefully designed to promote parent/child interactions about the behaviors. For example, in a specific “Do” week parents are provided with a set of “SWITCH Activity Cards”. Each card is detailed with activity name, necessary equipment and space, and description of activities that the child can do on their own or with their parent. In this round of formative evaluation, the SWITCH® program was implemented in the same manner for the print and the online conditions. The parents in the print group received booklets for each month of the program and the same resources were released on the website for parents to browse, print, and use.

The key behavior change strategy in SWITCH® is self-monitoring as children are tasked to work with their parents to complete the weekly SWITCH® Tracker sheets. The Trackers prompt children to choose a daily behavior goal (e.g., 60 minutes of physical activity in a “Do” day/week), record the actual behavior result (e.g., actual activity time per day), then tally and add up the points earned if goal is attained (e.g., 3 points/day and 21points/week maximal). Children turn in the Trackers each week to accumulate SWITCH® points which are redeemable for various incentives throughout the program. District program managers hired by the local YMCA made weekly site visits to gather the Trackers and to provide the weekly and monthly incentives for participation.

The link between the school and the home is an important component in the SWITCH® program since it keeps parents more involved in the program. The previous efficacy-based studies were able to systematically implement the SWITCH® program with optimal fidelity since research assistants could handle the coordination and communication. However, anecdotal observations of current SWITCH® schools indicate that there is considerable variability in the degree of school engagement. To enable broader dissemination of the program it is important to better understand the impact of school engagement on the program outcomes. While not a planned part of the study, school engagement was included as a moderating variable in the analyses.

### Evaluation framework and measures

The evaluation of the SWITCH® program was guided by the established PRECEDE PROCEED Model [18]. There are a number of advantages of this model for the present study and for the subsequent dissemination efforts. One key advantage is that it is consistent with the social-ecological approaches that underlie the SWITCH® intervention. The “*Epidemiology Assessment*” in the PRECEDE Phase challenges the planner to separate out the behavioral and environmental factors that are targeted in the intervention. The subsequent “*Educational and Ecological Assessment*” then splits these influences into Enabling, Reinforcing and Predisposing Factors. In SWITCH®, parents are viewed as Enabling Factors since they enable, facilitate and promote behavior change in their children. Schools are viewed as Reinforcing factors since the teachers are positioned to remind and reinforce the systematic efforts with the program. The goal of the programming is to facilitate self-monitoring and behavioral skills in children and these are considered the key Predisposing Factors. The final stage of the PRECEDE phase (*“Administrative and Policy Assessment”*) captures variables thought to be potentially important for the implementation and sustainability of the program. Examples in the present study include school characteristics and school engagement. A second advantage of the model is that it incorporates an evaluation of the process, the impact (i.e. the intervention itself), and the final outcome in the Proceed phase. The structure of this model has been endorsed as an appropriate model for dissemination and implementation research [19]. Details of the process, impact and outcome variables examined in the evaluation are summarized below.

#### Process measure (Child Involvement)

The primary goal of the program was to promote self-monitoring and goal setting for changing diet, activity and screen time behaviors. Children (with parental help) were tasked with filling out SWITCH® Trackers each week and bringing them to school for incentives (points redemption). Therefore, the percent of children completing and returning SWITCH® Trackers was viewed as the key process measure. Another key process measure was the overall parent satisfaction with the SWITCH® materials. Parent satisfaction was captured with a single multiple-choice item anchored on a 4-point Likert scale ranging from 1 = “very satisfied” to 4 = “very dissatisfied”.

#### Impact measures (Parent/Child Interactions)

The focus of the SWITCH® programming is to facilitate parent/child interactions about healthy lifestyles. The impact of the programming was assessed with items capturing parent’s report of the quality of interactions related to the three target behaviors (“Do, Chew, and View”). For example, the item for “Do” behavior was stated as: “Did SWITCH help you talk to your child about being physically active?” The answers included 1 = yes, helped a lot”, 2 = “yes, helped somewhat”, 3 = “yes, helped a little”, and 4 = “no, did not help”. The mean of the three items was used to reflect the overall impact of the SWITCH® program on parent/child interactions.

#### Outcome measures (Child Behaviors)

Children’s weight management behaviors were measured by 8 items, with 2 items each for the Do and View behaviors, and 4 items for the Chew behavior. Parents were asked to compare their child’s current (upon intervention) behaviors to before participating in SWITCH®. An item for the chew behavior is stated as “Compared to before your family participated in SWITCH®, does your child consume fruits?” The answers ranged from 1 = “a lot less often” to 5 = “a lot more often”. Other Chew behavior items asked about children’s consumption of vegetables, 100% fruit juice, and soft drinks. The means for each of the individual behaviors was used for the analyses.

#### Moderating variables (Teacher/School Engagement)

The degree of teacher/school engagement was assessed as a moderating variable using the numerical ratings conducted by two experienced program managers who had worked as SWITCH® staff for over six years. The program managers rated each teacher’s level of engagement from 1 (low) to 3 (high) based on their in-person field observations and interactions with each school. There were a total of 22 teachers (ranging from 1 to 3 teachers per school; median =3) being rated by the program managers. The researchers then averaged the scores for the teachers of the same school and categorized two levels of teachers’ engagement for data analysis: 1 = “low” (≤mean), 2 = “high” (>mean).

### Data analysis

The focus of the evaluation was on direct comparisons of process, impact and outcome measures between the print and online versions of SWITCH®. For the process evaluation, we compared the rate of completion of the Trackers between print and online using graphic techniques, and between the highly engaged schools and the lowly engaged schools using descriptive analysis (Mean and Standard Deviation). It was not possible to examine these data using statistical methods since the sample sizes for the school-related outcomes were too small. We also descriptively compared the level of parental satisfaction (i.e., average % for “very satisfied” and “satisfied” parents) with the SWITCH® program between the print and online groups. Standard analytic techniques described below were used to examine the Impact and Outcome measures. Frequencies of key measures were first reported to provide an overall sense of parent reactions to the programming. Descriptive statistics (mean and standard deviation) were provided for the primary measures and these were reported for both print and online groups as well as combined. Two-way multivariate analyses of variance (MANOVAs) were used to statistically examine differences in Impact and Outcome measures between the print and online groups and between lowly and highly engaged schools. Homogeneity of variances test was performed prior to the series of inferential statistical analyses.

## Results

The SWITCH® programming was carried out as planned and the return rates of the SWITCH® Trackers (obtained from the district program coordinators each week) provided valuable process data about the programming. Surveys were completed by 91 parents (43% return rate) and these data were used to capture impact and outcome measures. The majority of respondents on the survey were female (81%) and return rates were slightly higher from the print schools (n = 50, 55% of respondents) compared to the online schools (n = 41, 45% of respondents). All impact and outcome variables on the survey had low skewness and kurtosis absolute values (absolute values ≤ 2.0), indicating no violation of normal distribution. The parent participants reported similar mean scores for these variables between the two SWITCH® versions. Levene’s homogeneity of variances test revealed that all variables had homogeneous variances between groups (p > .05).

Overall, parent satisfaction was high with 93.2% of parents reporting being “satisfied” (70.5%) or “very satisfied” (22.7%) with the SWITCH® program. Most parents (68.2%) reported that they spent less than an hour per week on SWITCH® activities. However, more than a third (35.7%) reported that their child spent more than 2 hours per week on the materials. No significant differences were observed in mean parent satisfaction scores between print and online (Cohen’s d = .09). The district program managers rated 6 lowly engaged schools and 4 highly engaged schools. Reports from the SWITCH® trainers revealed large differences in the degree of school engagement across the sites. Variability in school engagement can have a profound effect on outcomes so school engagement was studied more directly as a moderating variable.

### Process evaluation

**Figure 1:**
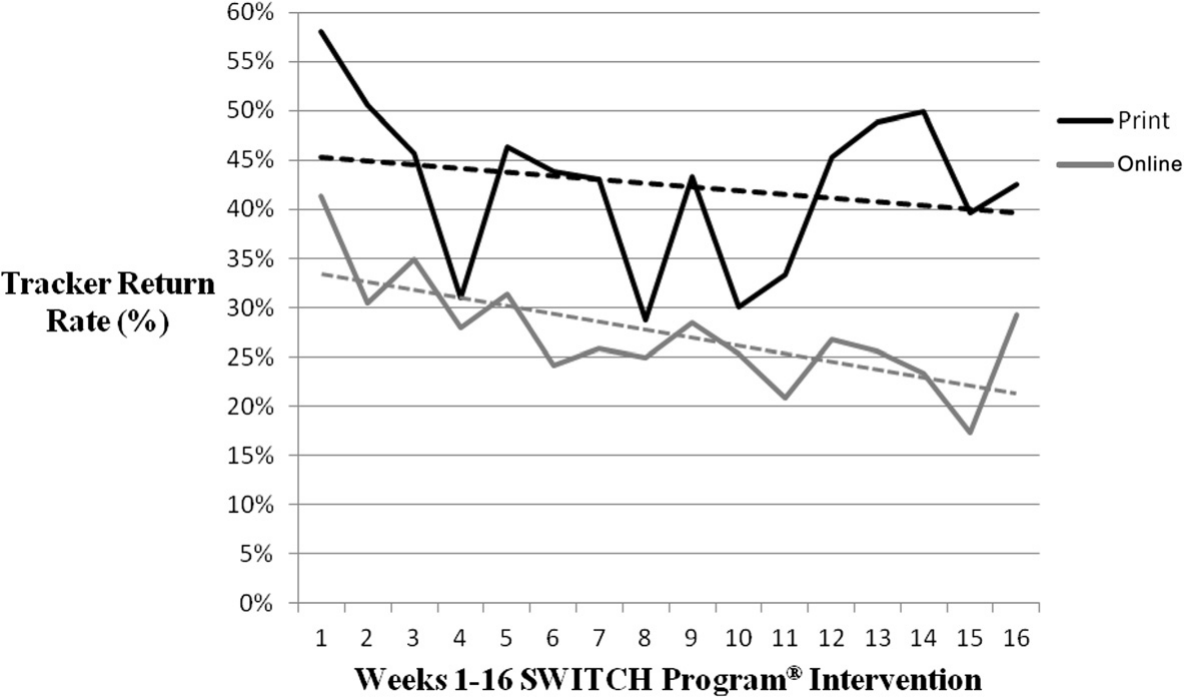
Weekly (Week 1–16) Tracker return rate throughout the SWITCH® Intervention. Both print and online groups witnessed a weekly decline trend for Tracker return rate, but the online group had a greater decline.

**Figure 2:**
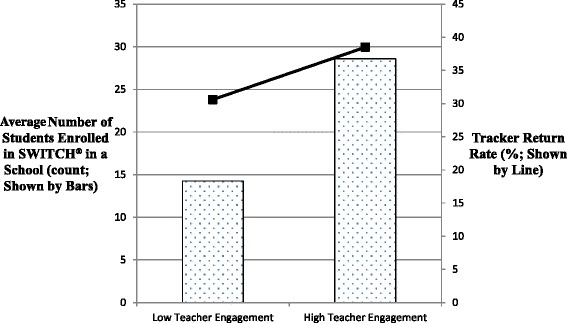
School enrollment size (left Y-axis) and Tracker return rate (right Y-axis) between schools with lowly and highly engaged teachers. The higher level of teacher engagement was associated with higher student enrollment in the SWITCH® program and higher Tracker return rate.

The key process measure in the study was the return rate of the SWITCH® Trackers. The Trackers serve as the primary behavior change tool in the program since it necessitates direct parent/child interaction and self-monitoring of their lifestyle. The overall return rate was 34.1% ± 13.0% across the 16 weeks of the programs but rates were higher in the print schools (42.5% ± 11%) than the online schools (27.4% ± 10.9%). Involvement in the program diminished across the course of the program for both groups as evidenced by declining return rates over time (Figure 1). Examination of the data revealed wide disparities in the Tracker return rates across the schools. To examine this, schools were categorized into two groups based on the mean score on the teacher engagement ratings (mean = 2.09). Figure 2 shows the average Tracker return rate and student enrollment size between the schools with lowly or highly engaged teachers. The results showed that the Tracker return rates were considerably higher in schools that were rated as highly engaged (mean rate = 38.5% ± 13.3%) compared with those rated as lowly engaged (mean rate = 28.6% ± 11.9%). The highly engaged schools had higher enrollments (mean enrollment size = 30.6 ± 6.9) than the lowly engaged schools (mean enrollment size = 14.3 ± .5) so this likely contributed to the differences in parent/child involvement.

### Impact and outcome evaluation

The impact variables capture the degree of parent/child interactions about the target behaviors. The majority of parents reported that the program helped them (either “a lot” or “somewhat”) in talking to their child about being active (68.9%), eating healthy (71.1%) or watching less TV (64.7%). The descriptive results (mean and SD) for these three impact variables are shown in the top portion of Table 1 for both the print and online schools (as well as for overall). The outcome variables capture the parents’ perceptions of behavior change resulting from the programming. The majority of parents reported favorable changes in physical activity habits (68.5%), eating habits (62.2%) and media habits (67.4%) at the end of the program. The descriptive results of the parent-reported behaviors are shown in the bottom portion of Table 1 for the print and online schools (as well as combined). The two-way MANOVA revealed no significant difference in the impact (i.e., interaction with Do, View, and Chew parts of the SWITCH® program) and outcome measures (i.e., parent-reported child Do, View, and Chew behaviors) between versions (Wilks’ Lambda = .93, F_6,76_ = 1.01, p = .43) or across levels of teacher engagement (Wilks’ Lambda = .88, F_6,76_ = 1.76, p = .12). The interaction was also not significant (Wilks’ Lambda = .90, F_6, 76_ = .1.34, p = .25). The effect sizes for the impact and outcome measures between print vs online SWITCH® programming were generally low (Cohen’s d ranged from -.12 to .40; See Table 1).

**Table 1:** **Parent/Child interactions with SWITCH® program and parent-reported “Do”, “View”, and “Chew” behavior outcomes**

**Variables**	**Overall**			**Print SWITCH®**			**Online SWITCH®**			**Cohen’s d**
	**N**	**M**	**SD**	**n**	**M**	**SD**	**n**	**M**	**SD**	
**Impact Measures (Parent/Child Interaction)**										
with SWITCH® “Do” part	90	2.96	.90	49	2.98	.92	41	2.93	.88	.06
with SWITCH® “View” part	88	2.81	.87	48	2.81	.91	40	2.80	.82	.01
with SWITCH® “Chew” part	90	3.03	.92	49	3.10	.90	41	2.95	.95	.16
**Outcome Measures (Behavior Outcomes)**										
“Do” behavior	89	3.73	.70	48	3.84	.64	41	3.60	.74	.35
“View” behavior	89	3.69	.65	49	3.65	.62	40	3.73	.70	-.12
“Chew” behavior	90	3.64	.59	49	3.75	.64	41	3.52	.51	.40

*Note*: SWITCH® “Do”, “View”, and “Chew” refer to ≥ 60 minutes moderate-to-vigorous physical activity per day, ≤ 2 hours screen time per day, and ≥ 5 servings of fruits and vegetables per day, respectively.

## Discussion

The present study was designed to formatively evaluate the relative utility of a print-based program of SWITCH® and an identically configured web-based version. Since participants in the print and online conditions received identical treatments and incentives, any differences can be attributed to the form of interventions received during the program. It was hypothesized that the online version would work similarly to the more expensive and less-sustainable print version. The comparison between the two versions of SWITCH® program was made by examining differences in the designated process, impact, and outcome measures.

The process evaluation revealed differences in the absolute numbers of SWITCH® trackers returned between the conditions. This makes sense considering that the print version allowed parents to simply tear out the pre-printed Tracker and fill it out. In the online version, parents had to print materials themselves before engaging with the program. The results confirmed a plausible and somewhat expected weakness of the online materials; however, a closer examination of the data revealed that school engagement explained some of the differences in both school enrollment and return rates. Specifically, schools rated as highly engaged had much higher class enrollments and much higher Tracker return rates than schools rated as lowly engaged by the district coordinators. This explains at least some of the observed differences since engagement and class sizes were higher in the print versus online SWITCH® schools. The results suggest that teacher/school engagement may have a moderating influence on parent recruitment and participation in the SWITCH® program. However, it is also possible that the Tracker rates and engagement were higher in schools that had higher enrollments. For example, if there are more kids participating, teachers may pay more attention to the program and this would, in turn create more interest in the students. Thus, it is not completely clear whether school engagement is a cause or a consequence of child involvement. Regardless of the mechanism, it is clear that building school and teacher support is an important consideration for effective implementation of the SWITCH® program. This result shows the importance of coordinating school personnel and parents and creating an ecological environment for children to enhance self-monitoring with their health-related behaviors [20].

It is noteworthy, and somewhat expected, that weekly Tracker return rates declined trend over time since it is typical for participants to lose interest in programming over time. This was evident in both conditions, but suggests the need for creative strategies to maintain parent/child interest in SWITCH®. The trend was noted during implementation and the research team worked with the district coordinators to pilot a peer incentive system in the last month that would provide double incentives if peer-selected partners both returned their Trackers. This strategy is consistent with behavioral economics theory and previous research suggesting that peer incentives may help hold youth accountable [21-23]. We noted higher average Tracker return rates in month 4 compared to the month 3, suggesting that this strategy may have value in future programs. This finding is consistent with perceptions from some parents that the program was too long; however, it may also reflect a need for more innovative strategies or challenges to maintain participants’ motivation over time. For example, the SWITCH® research team could incentivize participants using disseminating stickers as informational stimuli or providing additional incentives to individuals or groups of participants for achieving higher goals (group incentives). Additional work is clearly needed to maintain interest and involvement over time.

While we observed some differences in Tracker returns between conditions (and declines over time) there were no significant differences in the key Impact or Outcome variables. This indicates no difference in parent-reported SWITCH®-related interactions with their child and no differences in the observed behavior changes. These are important results since they demonstrate that the web-based platform is as effective as the more expensive print-based materials in promoting parent/child engagement. It is especially noteworthy considering that differences were evident with the return of Trackers. This suggests that the online materials may have had other subtle benefits on parent/child interactions not captured by the Trackers. With easier access to the internet in the modern era, health-enhancing interventions have started to move to the virtual world [24-29]. Web-based interventions often allow more interactivity for the users, but they may not be fully accessible for all [29]. A recent study that examined strategies to reduce multiple behavioral risk factors found that nearly equivalent number of participants chose to use print or web-based materials, while being offered the two options [29]. The users of web-based materials in the intervention tend to be younger adults with greater computer comfort and more frequent use of internet on the daily basis [29]. The online SWITCH appeared to be well-received by the parents of young children in this current study. It confirms that web-based childhood obesity prevention programs may have good potentials to impact participants’ interaction with the programs as well as their healthy behaviors. It is also noteworthy that we observed no differences in impact or outcome measures between levels of school engagement. This suggests that parent interactions can be promoted somewhat independently of strong school support. Ultimately, we would expect to have optimal effectiveness in schools that actively promoted and utilized aspects of the SWITCH® materials as part of their school-based lessons since this would provide a more integrated and coherent message.

The formative evaluation supports the utility of the web-based platform but it is clear that the current version of the website does not provide considerable value. This is because parents still had to print copies of the Trackers as well as other SWITCH® materials and submit them manually. It was important to test this basic system first to show that it at least produced similar or equivalent outcomes. Nevertheless, web-based programs are supposed to provide a higher level of interactivity than the print materials to actively engage the users [29]. The online version of SWITCH tested in this current study, however, had limited interactivity. Thus, a more comprehensive and interactive web-based platform needs to be established to enable features such as direct logging of Trackers online by parents and automated recording and monitoring of completions by the program leaders. The planned enhancements to the content management for SWITCH® will enable school programmers to manage communication and to take advantage of other web attributes (e.g. ability to link to web resources and social media). Parents would also have an easier time interacting with the program and their child since they would be able to directly track progress using email/web tools that are common in contemporary society. Thus, we expect that interactions and engagement can be dramatically increased.

An important observation in the present study is that the degree of school/teacher engagement had an important impact on enrollment and involvement in the program. This is a common and somewhat expected finding in effectiveness research [30] and additional work is needed to better understand the factors influencing motivation and engagement at the organizational level. The earlier versions of SWITCH® were able to directly control and manage the school environment but this is not possible in more distributed effectiveness or dissemination studies. The plan for future development is to build in training and support to increase school engagement. Complementary SWITCH® materials for PE and classroom teachers will provide additional intervention while also directly reinforcing the knowledge and skills that children learn at home. Similar SWITCH® visibility in nutrition education programs could possibly enhance parenting efforts to promote healthier eating. The results of the present study demonstrate that school leaders/coordinators can be trained to facilitate the basic dissemination of the SWITCH® program in their school. It is likely that additional school modules would provide even larger benefits so this is a priority for future research.

### Limitations

Despite the encouraging findings, this formative evaluation was limited by the small sample and the lack of pre-test measures. While most of the parents-children dyads contributed data to the earlier efficacy study [9], only about one third (34.3%) of parents completed the post-intervention survey in this less controlled evaluation. However, no violation of statistical assumptions was observed in all inferential analyses so the results are defensible. Another potential limitation is the inability to directly link parent reports to the child’s engagement. The survey responses were all anonymous to allow the study to be viewed as exempt by the IRB and this limited our ability to examine the extent to which parent involvement influences individual behavior. These issues will be explored in supplemental studies.

## Conclusions

In summary, the formative evaluation demonstrates the viability of the online SWITCH® program as a more cost-effective method for dissemination. The online version yielded similar results for both the impact and outcome measures. Thus, parents and children were able to interact with the program in a similar fashion, regardless of the versions received. The differences in Tracker rates may not be relevant since we ultimately envision a system in which the participants would log SWITCH® trackers directly on the website and see accumulated points over time. The school coordinator could also view results for individuals or classes without having to laboriously track the printed sheets. Thus, the reported differences in Trackers returns may not be a major concern, when the new web platform is developed.

The formative evaluation study shows that the online SWITCH® program holds great potential as an efficacious and sustainable childhood prevention program. The findings will guide the development of the more robust SWITCH® platform as well as the additional school-based modules (i.e., SWITCH® PE, SWITCH® Classroom). It is expected that the refined SWITCH® content management system will be able to more effectively impact children’s adoption of healthy lifestyles in school. The modularized system will facilitate integration of classroom, PE and lunchroom components and enable school wellness teams to carry out programming on their own. The SWITCH program provides a model consistent with CDC recommendations for Comprehensive School Physical Activity Programs (CSPAP).

## Abbreviations

ANOVA: Analysis of variance
PE: Physical education
TV: Television
